# Role of anti-vimentin antibodies in allograft rejection

**DOI:** 10.1016/j.humimm.2013.06.006

**Published:** 2013-11

**Authors:** Marlene L Rose

**Affiliations:** National Heart and Lung Institute, Imperial College, Harefield Hospital, Harefield, Middlesex UB9 6JH, UK

**Keywords:** AMR, antibody mediated rejection, AVA, anti-vimentin antibodies, CFA, complete Freund’s adjuvant, HMEC, human microvascular endothelial cells, MMF, mycophenolate mofetil, PAF, platelet activating factor

## Abstract

Production of anti-vimentin antibodies (AVA) after solid organ transplantation are common. Although classically thought to be expressed mainly within the cytosol, recent evidence demonstrates that extracellular or cell surface expression of vimentin is not unusual. This review examines the evidence to assess whether AVA contribute to allograft pathology. Clinical studies suggest that AVA are associated with cardiac allograft vasculopathy in heart transplant recipients. Studies in non-human primates confirm that production of AVA after renal and heart transplantation are not inhibited by Cyclosporine. Experimental studies have demonstrated that mice pre-immunised with vimentin undergo accelerated acute rejection and vascular intimal occlusion of cardiac allografts. Adoptive transfer of hyperimmune sera containing AVA into B-cell-knock-out mice caused accelerated rejection of allografted hearts, this is clear evidence that antibodies to vimentin accelerate rejection. AVA act in concert with the alloimmune response and AVA do not damage syngeneic or native heart allografts. Confocal microscopy of allografted organs in vimentin immunised mice shows extensive expression of vimentin on endothelial cells, apoptotic leukocytes and platelet/leukocyte conjugates, co-localising with C4d. One explanation for the ability of AVA to accelerate rejection would be fixation of complement within the graft and subsequent pro-inflammatory effects; there may also be interactions with platelets within the vasculature.

## 1 Introduction

Autoantibodies to vimentin (AVA) are commonly produced by patients with autoimmune diseases such as Lupus and rheumatoid arthritis [1,2], they are also found after solid organ transplantation [3–5]. Vimentin is an intermediate filament protein found in cells of mesenchymal origin, hence it is expressed within the cytosol of adult leukocytes, fibroblasts and endothelial cells. However, it can be expressed on the surface of cells and be secreted under certain conditions making it of interest as an antigen which can elicit an immune response. The evidence suggests that production of autoantibodies to vimentin reflect tissue damage, but whether anti-vimentin antibodies accelerate or accentuate tissue damage is less certain. The purpose of this review is to describe the distribution of vimentin within tissues and organs and assess the evidence from clinical and experimental studies that autoantibodies to vimentin contribute to allograft pathology.

## 2 Distribution and isoforms of vimentin

The most abundant common form of vimentin, detected on reducing gels is a 55-kDa molecule, representing intermediate filaments. Vimentin is composed of three domains; the amino-terminal domain (head domain), the central core (rod domain) and the carboxy-terminal domain (tail domain). Vimentin is expressed on the cell surface of apoptotic T cells [6] and neutrophils [7]. Using monoclonal antibodies to the different domains of vimentin, it has been determined that the tail domain (reacting with the V9 antibody) is exposed on apoptotic neutrophils [7], while both rod and tails are expressed on the surface of apoptotic T cells [6]. The molecule has several cleavage sites for caspase 3 and caspase 8 and caspase-dependent cleavage of vimentin is an essential pre-requisite for apoptosis [8]. During apoptosis nuclear and cytosolic antigens become disorganised, resulting in exposure of cryptic epitopes [9], raising the possibility that apoptotic cells act as reservoirs of autoantigens [10]. In view of the fact that apoptosis accompanies many stages of allograft rejection, there is the possibility that apoptotic cells stimulate production of autoantibody to vimentin. In addition, vimentin is expressed on the cell surface of activated platelets and is secreted by activated macrophages [11,12]. Further evidence of cell surface expression and secretion of vimentin was provided by Xu et al [13]. Xu et al demonstrated that the monoclonal antibody Pal-E, used for many years as a marker of endothelial cells, recognises a high molecular weight form of vimentin, 120 kDa, within and adjacent to vesicles near the luminal surface of Human Microvascular Endotheial Cells (HMEC). Pal-E did not stain the intermediate filaments of the HMEC. The authors performed further experiments which suggest the extracellular and secreted form of vimentin is formed as a result of post-translational protein modification. These authors also demonstrated that cultured HMEC secrete Pal-E reactive vimentin, of both 55 kDa and 120 kDa, and that Pal-E reactive vimentin is found in human plasma. The biological function of cell-surface and secreted vimentin found in blood is not known, but evidence suggests that vimentin may regulate movement of circulating lymphocytes [13–15]. However, others have demonstrated that Pal-E reacts not with vimentin but with plasmalemmal vesicle 1 (PV-1) also called fenestrated endothelial-linked structure protein (FELS) [16] and Jaalouk et al have determined that this antibody reacts with an epitope of human neuropilin-1 (NRP-1) found in endothelial cells [17]. Bilalic et al have reported that patients on dialysis make AVA to the 49 and 60 kDa isoforms and that activated human T cells express the 49 kDa isoform [18]. These authors suggest that different isoforms may be expressed by activated or damaged cells within organ allografts. Post-translational modification of vimentin caused by oxidative stress or citrullination will result in a molecule with a different structure to the native molecule, and hence likely to be recognised by the immune system not as an auto-antigen but as heterologous protein. Hence assaying antibodies to citrullinated -vimentin is a more sensitive assay for measuring disease severity of rheumatoid arthritis than assaying antibodies to native vimentin [19]. The studies descried above demonstrate the heterogeneity of vimentin within different tissues, likely caused by post-translational modification. These studies also suggests that AVA produced by different patients recognise different antigenic epitopes of vimentin, determined by the cellular source of the vimentin.

A number of studies have investigated expression of vimentin on organs before and after transplantation. Immunocytochemistry of human and non-human primate hearts has demonstrated that vimentin is expressed within endothelial cells and it is strongly expressed on the luminal surface of blood vessels showing graft vasculopathy [3], isoforms were not investigated. This reflects endothelial expression of cell-surface vimentin and also probably reflects its presence in proliferating/migrating smooth muscle cells. Vimentin is not expressed by adult cardiomyocytes. In kidneys, normal tubular cells are negative for vimentin, but during rejection the cytosol of some tubular epithelial cells become vimentin positive [4]. Similarly, the cytosol of reactive, regenerative or apoptotic renal tubular cells are vimentin positive [20]. Renal interstitial cells constitutively express vimentin, and expression increases during rejection [4] and ischaemic injury [21]. Further investigations of the isoforms and the precise cellular localisation of vimentin before and after rejection would be informative.

## 3 Clinical studies

It was initially shown in 2001 that heart transplant patients produce IgM anti-vimentin antibodies within 2 years of transplantation and that higher titres in the first two years were predictive of cardiac allograft vasculopathy (CAV) at 5 years [5]. In this study IgM antibody was detected but not IgG antibodies and none of the patients had AVA prior to transplantation. Nath et al have also reported significantly higher levels of antibodies to vimentin in patients who develop CAV [22], but whether these were IgM or IgG antibodies was not reported. These authiors did not investigate pre-transplant levels of AVA. An increased frequency of CD4+ T cells that secrete IL-17 in response to vimentin was also found in patients with CAV along with a decrease in IL-10 secreting cells, indicating breakdown of tolerance to vimentin in patients with CAV [22]. Barber et al using A^∗^02:01 vimentin binding tetramers, detected self-restricted CD8+ T cells which bound to vimentin tetramers in eight cardiac transplant recipients [23]. Together these results demonstrate breakdown of tolerance to vimentin and development of autoreactive vimentin specific CD4+ and CD8+ T cells in cardiac transplant recipients.

Studies in clinical renal transplantation have demonstrated that patients make AVA after renal transplantation [24]. Carter et al demonstrated that recipients of kidneys from non-heart-beating donors (with long ischaemic times) produced significantly higher levls of AVA at 1 month and 6 months than recipients of kidneys from heart beating donors (with shorter ischaemic times). A recent study of renal transplantation has suggested that some patients have IgG AVA prior to transplantation and these patients are more likely to develop chronic graft vasculopathy (Besarani D, Cerundolo L, Smith JD, Procter J, Barnado MCN, Roberts ISD, Friend PJ, Rose ML, Fuggle SV, in preparation). Studies of the factors which influence production of AVA prior to renal transplantation would be of interest. Bilalic et al performed a proteomic study of sera from 28 patients on dialysis awaiting renal transplantation [25] and discovered many made autoantibody. Autoantibodies to the 60 kDa and 49 kDa isoform of vimentin were amongst the most common, suggesting that prolonged dialysis’ may be a risk factor for AVA. Our own extended studies of patients awaiting thoracic organ transplantation (350 heart and 458 lungs, during the period 1998–2012) detected only 10 and 6%, respectively of patients with pre-transplant IgM AVA, IgG were not sought (Rose ML, Smith JD unpublished data). However, it maybe that patients undergoing re-transplantation have AVA, as may patients with pre-exisiting autoimmune diseases.

Nath et al have reported that production of AVA in cardiac transplant recipients is associated with AMR in the first year after transplantation [22], and that these antibodies preceded diagnosis of AVA by several months. Our own studies of AMR in heart transplant patients investigated 17 patients with late AMR (Smith JD and Rose ML, unpublished); in 15 cases donor specific HLA antibodies were found at the time of diagnosis of AMR and in two patients IgG AVA were found at the same time. Whereas it is not surprising to find AVA concomitantly with donor specific antibodies to HLA, it is of more interest that In two patients, IgG AVA were detected at the time of diagnosis of AMR, in the absence of donor specific HLA antibodies. The possibility that other autoantibodies (which were not investigated) contributed to AMR cannot be excluded.

## 4 Effects of immunosuppressive drugs

Studies in non-human primates confirm production of AVA is associated with solid organ transplantation and is not sensitive to Cyclosporine. Azimzadeh et al studied heart-transplanted cynomolgus monkeys and demonstrated that IgM/IgG AVA were elaborated within 30 days in unmodified acute rejection and similarly produced in animals treated with Cyclosporine [3]. CD154 blockade did not prevent production of IgM AVA but did delay the appearance of IgG AVA. It is interesting to note that elution of antibodies from hearts of monkeys with CAV were shown to contain AVA as measured by ELISA, but hearts from normal monkeys did not contain AVA. Imunocytochemical examination of transplanted hearts from cyomolgus monkeys with positive IgM AVA titres showed presence of C4d in cardiac biopsies, in the absence of detectable alloantibodies, suggesting that IgM AVA fix complement. Jonker at al studied AVA in rhesus monkeys following renal transplantation [4] and also demonstrated production of AVA was very common, occurring in 31/37 animals and was not inhibited by Cyclosporine but was partially inhibited by CD40 and CD86 blockade. In both studies elaboration of AVA was associated with graft vasculopathy, although some animals developed graft vasculopathy in the absence of detectable AVA. Hence AVA are a contributing factor in the pathogenesis of chronic rejection, either indirectly as a marker of tissue damage or directly (mechanisms described below). Studies in heart transplant recipients have also demonstrated different sensitivities to immunosuppressive drugs, hence production of IgM AVA was significantly less in patients receiving myocophenolate mofetil (MMF) than those receiving azathioprine [26]. Production of HLA antibodies were also reduced by MMF.

## 5 Insights into mechanisms from experimental studies

Immunisation of mice with two injections of recombinant mouse vimentin, one of them in the presence of Complete Freund’s Adjuvant (CFA) breaks tolerance to vimentin [27]. Such mice produce high titres of IgG and IgM AVA for 2–12 weeks, the predominant subclass of IgG being the complement fixing IgG2b isoptype. T cells from the spleens of vimentin immunised mice produced IFNg when re-stimulated with vimentin in vitro – demonstrating a strong Th1 response to mouse vimentin. When these mice were transplanted with hearts differing at multiple minor histocompatibilty antigens (129/sv donors into C57BL/6 recipients, transplanted 7 days after the booster injection of vimentin) there was accelerated rejection in vimentin immunised mice compared to mice immunised with control protein hen-egg lysozyme (8.4+/− 1.5 days compared to 13.3+/− 2.2 days, *p* = <0.0001). Transplantation of syngeneic hearts or isografts (from C57BL/6 donors) into vimentin immunised C57BL/6 recipients did not result in rejection. The minor histocompatibility combination was chosen because C57BL/6 mice do not make alloantibodies to 129/sv donors [27], hence presence of C4d in the hearts can be attributed to AVA. C4d was found in the hearts of vimentin immunised mice rejecting 129/sv hearts, as was presence of P-selectin on endothelial cells. Significantly more CD41 platelets and T cells were found in grafts of vimentin -immunised mice and AVA were eluted from rejected hearts. Adoptive transfer of hyperimmunised sera from vimentin-immunised rabbits into B-cell knock-out mice bearing 129/sv hearts resulted in accelerated rejection, demonstrating a clear role for AVA in rejection of cardiac allografts [27]. It is important to note that AVA alone are not sufficient to cause graft damage, isografts continued to beat for 90 days in the presence of high titre of IgG AVA brought about by repeated immunisation of recipients with vimentin following transplantation; isografts at the end of this period did not show any signs of myocyte damage or graft vasculopathy. In this respect, the anti-vimentin response is unlike the autoimmune response to cardiac myosin [28,29] or collagen [30] which causes damage to isografts. Confocal microscopy of rejecting allografts in vimentin immunised mice showed much higher levels of exposed vimentin than isografts [27]. In isografted hearts, vimentin expression was low at day 2 and was confined to some leukocytes and endothelial cells, in allografts there was extensive expression of vimentin on apoptosing leukocytes and platelet-leukocyte conjugates co-localising with C3d deposition. There was also expression of vimentin on non-apoptotic endothelial cells co-localising with C3d deposition. It seems likely therefore that damage by AVA is caused by retention of complement fixing AVA within allografts, to apoptotic cells, activated platelets and endothelial cells. Deposition of C4d and C3d are highly inflammatory and will accentuate the inflammatory response [31]. There have been no clinical studies investigating whether the isotype of IgG antibodies made to vimentin are the complement fixing isotypes (IgG3 and IgG1 in humans). However after cardiac transplantation the large majority of AVA are IgM, which fix complement.

## 6 Interaction with platelets and graft vasculopathy

In vitro studies have demonstrated that normal blood treated with patient sera containing high AVA IgM titres or with a vimentin specific monoclonal IgM leads to activation of platelets demonstrated by induced expression of P-selectin, fibrinogen, tissue factor and formation of platelet-leukocyte conjugates [32]. Normal blood would not be expected to contain activated platelets and in these studies it was demonstrated that AVA did not bind to platelets in the blood but to the 10% of leukocytes in normal blood that express vimentin on their cell surface, causing release of Platelet Activating Factor (PAF) which then activated platelets. Activated platelets express vimentin [12], hence allowing further interaction between AVA and platelets. The interaction of AVA with neutrophils and platelets, produces a pro-thrombotic phenotype which may contribute to the pathogenesis of thrombotic events that occur in autoimmune diseases as well as the thrombotic events that are associated with allograft vasculopathy. Similarly it has been argued that HLA antibodies to MHC class I antigens (constitutively present on platelets) act to potentiate graft rejection via activation of platelets [33]. Two experimental models of cardiac allograft vasculopathy were used to demonstrate that pre-immunisation of recipients with vimentin in CFA results in accelerated occlusion of blood vessels with smooth muscle cells [34]. Transplantation of 129/sv hearts into non-immunosuppressed C57BL/6 represents a multiple minor mismatch and results in acute rejection in 12–14 days [27]. In order to attenuate acute rejection and allow cardiac allograft vasculopathy (CAV) to develop, it is necessary to partially deplete circulating T cells with monoclonal antibodies, this results in CAV detected at about 3 weeks after transplantation [34]. Two models of CAV were used, transplantation of 129/sv into T cell depleted C57BL/6 and transplantation of FVB hearts into non-immunosupressed DBA/1, in both cases this results in significant intimal occlusion of donor coronary blood vessels (CAV) at days 30 and days 18, respectively [34]. When mice had been immunised with mouse vimentin to achieve high IgM and IgG titres and transplanted when titres were high, intimal occlusion was significantly greater at the times studied [34]. Confocal microscopy of grafts with luminal occlusion demonstrated co-localisation of vimentin on the luminal surface of endothelial cells with C3d, and co-localisation of vimentin with CD45 leukocytes in the graft. Vimentin also co-localised with CD41 platelets which were expressing CD62P. Immunisation of recipients with vimentin also caused increased influx of T cells in the hearts of these recipients. It is unlikely that autoreactive T cells damage the hearts because autoreactive T cells are MHC restricted and hence will not recognise vimentin on graft cells; however, antibodies are not MHC restricted and can presumably recognise peptides on any surface. Similar to the explanation of pathogenic effects of AVA on acute rejection, it is proposed that AVA become retained within the allograft during chronic rejection due to endothelial cells and apoptosing leukocytes expressing extracellular vimentin. A pro-coagulant microvasculature is associated with graft vasculopathy in humans. Hence the presence of fibrin deposition [35] and depletion of tissue plasminigen activator in the microvessels are predictive of heart transplant patients who develop CAV [36].

## 7 Summary

Although classically considered to be only expressed in the cytosol as intermediate filaments, it is now clear that isoforms of vimentin are also expressed on the cell surface and secreted under certain conditions. Vimentin is abundantly expressed in tissue as a result of tissues damage, as demonstrated by clinical and experimental studies. Elaboration of AVA probably reflects breaking of tolerance to this self-antigen caused by excessive tissue damage within an inflammatory milieu; although it is also possible that antibodies are made to post-translationally modified proteins, which may not require breaking of tolerance. Experimental studies have shown that production of high titre IgG to vimentin on its own does not cause damage to the syngeneic hearts [27] or native hearts (Mahesh and Rose unpublished) – hence prior damage to the transplanted organ is necessary for AVA to accelerate rejection. Prior damage is likely to be due to the alloimmune response. The simplest explanation for the damaging effects of AVA is that AVA fix complement within the allograft and the presence of complement has a pro-inflammatory effect. There is also the possibility of AVA interaction with neutrophils and platelets which may lead to platelet activation and a thrombotic milieu within the vasculature of the allograft. Until now, the majority of investigators have used ELISA to whole vimentin molecules to detect AVA, more precise associations with pathogenesis may be found using more specific assays to vimentin isoforms or to modified vimentin.
